# Strategies and Methodologies for Improving Toughness of Starch Films

**DOI:** 10.3390/foods13244036

**Published:** 2024-12-13

**Authors:** Yiwen Yang, Jun Fu, Qingfei Duan, Huifang Xie, Xinyi Dong, Long Yu

**Affiliations:** 1 Institute of Chemistry, Henan Academy of Sciences, Zhengzhou 450002, China; yangyw@hnas.ac.cn (Y.Y.); fujun@hnas.ac.cn (J.F.); xiehf0213@hnas.ac.cn (H.X.); xinyidong@hnas.ac.cn (X.D.); 2 School of Material Science and Engineering, Zhengzhou University, Zhengzhou 450001, China; 3 School of Food Science and Engineering, South China University of Technology, Guangzhou 510640, China

**Keywords:** starch, phase transition, toughness, plasticizer, modification, reinforcement

## Abstract

Starch films have attracted increasing attention due to their biodegradability, edibility, and potential use as animal feed from post-products. Applications of starch-based films include food packaging, coating, and medicine capsules. However, a major drawback of starch-based films is their brittleness, particularly under dry conditions, caused by starch retrogradation and the instability of plasticizers. To address this challenge, various strategies and methodologies have been developed, including plasticization, chemical modification, and physical reinforcement. This review covers fundamental aspects, such as the microstructures, phase transitions, and compatibility of starch, as well as application-oriented techniques, including processing methods, plasticizer selection, and chemical modifications. Plasticizers play a crucial role in developing starch-based materials, as they mitigate brittleness and improve processability. Given the abundance of hydroxyl groups in starch, the plasticizers used must also contain hydroxyl or polar groups for compatibility. Chemical modification, such as esterification and etherification, effectively prevents starch recrystallization. Reinforcements, particularly with nanocellulose, significantly improved the mechanical properties of starch film. Drawing upon both the literature and our expertise, this review not only summarizes the advancements in this field but also identifies the limitations of current technologies and outlines promising research directions for future development.

## 1. Introduction

The invention of plastic is a double-edged sword, as it has significantly enhanced the quality of human life while concurrently engendering severe ecological pollution through plastic waste. The packaging industry stands out as one of the foremost driving forces behind the exponential proliferation of plastics usage; thus, transitioning towards sustainable alternative packaging materials derived from renewable resources should be embraced as a more circular and eco-centric approach. Consequently, an urgent imperative arises to foster novel materials that offer commensurate functionalities to plastic products but are environmentally benign.

Starch is a widely available natural polysaccharide found in all grain crops, offering several benefits such as low cost, biodegradability, biocompatibility, and ease of processing. Its natural and modified forms are commonly utilized as biodegradable packaging materials and bio-coatings, particularly for extending the shelf life of fresh agricultural produce [1]. Furthermore, starch, approved by the US Food and Drug Administration (FDA) for its biocompatibility, is used in various applications, including edible packaging, medicinal capsules, tablets, suppositories, implants, and stents [2]. For example, in the pharmaceutical market, compared to traditional gelatin capsules, plant-based capsules made from starch achieve better biocompatibility, can avoid the transmission of prions, and are acceptable by vegetarians and some religious people, with great market value.

When used as packaging materials for food and drugs, starch-based films generally offer low costs, good transparency, solubility, and biodegradability [3]. However, the brittleness of starch films is a significant weakness, as their mechanical properties are inadequate to maintain structural integrity and resist deformation and damage during subsequent processing, thereby greatly reducing their utilization efficiency. Furthermore, high-quality packaging materials require better water vapor barrier properties to ensure product quality [4], but the strong hydrophilicity of starch results in poor water barrier properties and hydrophobicity for starch-based degradable plastics, leading to inferior mechanical properties in wet environments. Additionally, due to hydrogen bond interactions between and within molecules, natural starch cannot be directly thermally processed using convenient and popular facilities used for traditional plastic processing like extrusion and blowing film. Starches must undergo plasticization treatment to form thermoplastic starch with desired plasticity [5].

Herein, developing strategies and methodologies for improving the toughness of starch films is decisive for the future perspective on practical application, and efforts have been devoted massively to conquering this challenge. This paper firstly presents the hierarchical structure of starch, serving as fundamental knowledge to enhance the comprehension of the underlying factors contributing to the distinctive properties exhibited by starch-based films. Secondly, the phase transition behaviors of starch under different treatments are reviewed, mainly focusing on the change in the interior hydrogen bond network within starch molecules, with both intrinsic and extrinsic factors influencing the molecular behavior discussed. Then, we comprehensively examine diverse strategies and methodologies aimed at enhancing the toughness of starch films. These include chemical modification, and compositing with nanocellulose and other hydrophilic polymers, in conjunction with fabrication techniques. Our objective is to systematically summarize the respective advantages and disadvantages of these strategies, offering foundational knowledge and inspiration to guide the development of innovative and effective approaches to fabricating advanced starch-based films tailored for food and drug packaging applications.

## 2. Resources and Microstructures of Starches

### 2.1. Resources of Starches

Starch is a vital component in numerous staple foods, including bread, pastries, and noodles. Beyond its role in food, starch is widely used in non-food applications such as paper production, textiles, and adhesives, and it is also employed as a component of bioplastics after undergoing chemical modification, plasticization, or gelatinization into thermoplastic material. The primary sources of starch are plants, particularly those with high carbohydrate content. The major sources include the following [3,6,7,8,9,10]:

1. Grains and cereals: common sources like wheat, corn, rice, barley, oats, and millet serve as the primary contributors to starch in the global diet.
2. Pseudocereal grains: crops such as quinoa, amaranth, buckwheat, and chia seeds provide additional starch-rich options.
3. Roots and tubers: potatoes, sweet potatoes, cassava, and yams contain significant starch reserves in their roots and tubers.
4. Legumes: mung beans, red beans, black beans, and lentils offer a moderate starch content alongside protein.
5. Seeds: plant seeds like mango, litchi, and avocado also contribute relatively high starch levels.
6. Fruits: although less rich in starch than grains or tubers, fruits such as bananas, apples, and pears still contain notable amounts.
7. Plant leaves: leaves like spinach and kale have lower starch content compared to root crops but can still serve as a source.
8. Algae: certain species, such as spirulina, also provide starch.

The molecular weight, granule size and shape, amylopectin-to-amylose ratio, and crystallinity, as well as lipid and protein contents, vary significantly, depending on the source of the starch. As a result, key properties such as gelatinization behavior, retrogradation tendencies, and film performance also exhibit corresponding variability [11].

### 2.2. The Multi-Scale Structure of Starch

**Figure 1:**
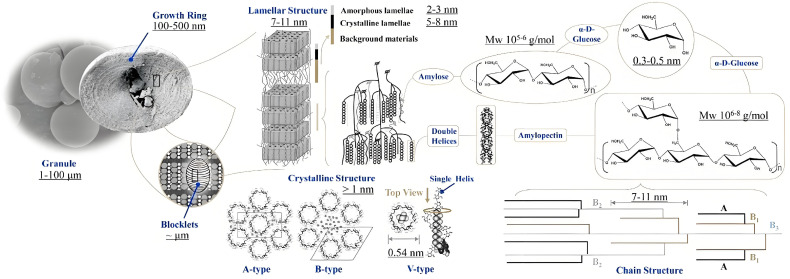
Schematic representation of hierarchical starch structures [12].

The microstructure of starch is highly organized and spans multiple hierarchical scales, as shown in Figure 1 [12]. Researchers have divided it into five levels, according to the scale from macro to micro dimensions, as known as granule morphology, growth ring, blocklets, lamellar structures (crystalline features), helical structures, and the amylose/amylopectin ratio [13,14,15,16]. Its two primary polysaccharides, amylose and amylopectin, form the basis of this intricate structure. Amylose, a mostly linear molecule, interacts with hydrophobic guest molecules to form V-type crystals. In contrast, amylopectin, with its highly branched structure, forms double helices that aggregate into either A- or B-type crystal. These crystalline regions, along with amorphous lamellae, assemble into lamellar structures that alternate between crystalline and amorphous layers. These lamellae, in turn, contribute to the semi-crystalline architecture of starch, which manifests as growth rings and blocklets.

The distinct molecular properties of amylose and amylopectin are critical to the functional behavior of starch. Amylose, composed of D-glucose units linked through α-(1,4)-glycosidic bonds, has a degree of polymerization (DP) ranging from 250 to 1000. The α-(1,4)-glycosidic bonds impart a natural twist to the glucan chain, allowing it to adopt a single-helical conformation. This helical structure readily forms complexes with various hydrophobic agents, depending on their size, which results in helices with six, seven, or eight glucose units per turn [17]. Amylopectin, on the other hand, features a backbone of α-(1,4)-glycosidic bonds with branches formed of α-(1,6)-glycosidic bonds. The branching chains in amylopectin are categorized by their length and degree of polymerization [18,19,20,21]. A chains: these unbranched chains, located at the outermost regions, have a DP of 6–12. B chains: classified as B1 (DP 13–24), B2 (DP 25–36), and B3 (DP > 36), these chains differ in their lengths and the number of clusters they span. C chains: each amylopectin molecule contains a single C chain with a reducing terminal residue. These molecular configurations and hierarchical structures of amylose and amylopectin provide starch with its unique physical and chemical properties, making it a versatile material for diverse applications.

Linear chains of A and B types with a degree of polymerization ranging from 10 to 20 have the tendency to form double-helical structures through hydrogen bonding, resulting in improved thermal stabilization and higher crystallinity. Each double helix is composed of six glucose residues per turn from each contributing strand, with a pitch of 2.1 nm and a length between 4 and 6 nm [22]. The periodic arrangement of these helical structures via intraregional hydrogen bonding leads to short-range order, which plays a crucial role in starch crystallinity, solubility, rheological properties, digestion, retrogradation, and gelatinization. The short-range ordered structure can be used as the basis for the formation of starch clusters, which mainly comprise A- and B-type short chains (DP < 36) [12]. Because of their shorter length and higher density, the short chains within the clusters are more likely to be arranged in an ordered method, which is the basis of starch crystallization [16].

**Figure 2:**
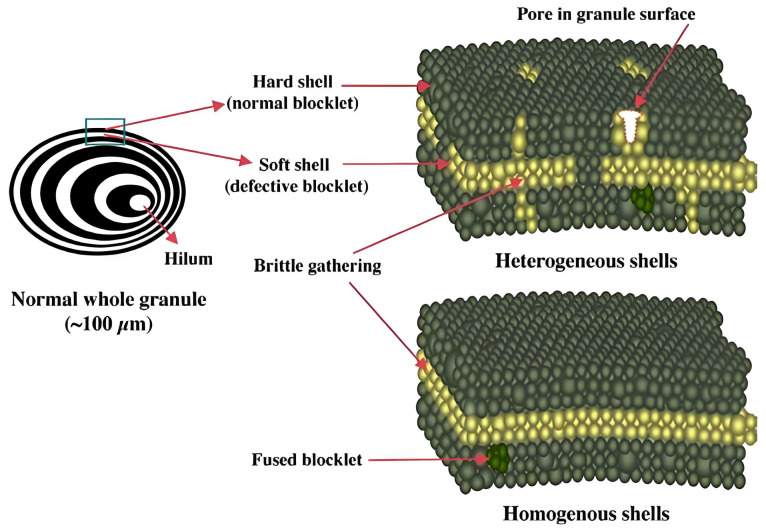
Scheme of starch granule structures [27].

In the subsequent stage of starch structure formation, approximately 150–300 double helices are densely packed into crystallites, which are categorized into A-, B-, C-, and V-type polymorphs [23]. The V-type polymorph arises from the aggregation of left-handed amylose single helices, typically after interaction with small ligands such as lipids and fatty acids [24]. The A-type crystalline structure is compact, with double helices tightly packed into a monoclinic unit cell (a = 20.83 Å, b = 11.45 Å, and c = 10.58 Å; space group B2) that contains eight inter-helical water molecules. By contrast, the B-type crystal features a hexagonal unit cell (a = b = 18.5 Å and c = 10.4 Å; space group P61) with 36 interstitial water molecules, resulting in a less dense arrangement [25]. The C-type polymorph, a combination of A- and B-type structures, is commonly found in certain legumes and grain starches [11]. Crystalline regions in starch organize into lamellae, which further aggregate into a super-helix structure with a pitch of 9 nm and a diameter of 18 nm, as evidenced in SAXS and WAXS diffraction studies [15]. These crystalline lamellae, along with amorphous lamellae, alternate to form semi-crystalline blocklets. Blocklets represent an intermediate structural level between growth rings and amylopectin lamellae [26]. Each blocklet incorporates the super-helix, which is thought to correspond to a single amylopectin macromolecule. Blocklets exhibit a spherical morphology with diameters ranging from 20 to 500 nm, varying depending on the starch source and the granule’s internal location [15]. Surrounding the blocklets is an interconnecting matrix referred to as the blocklet complex. Tang et al. introduced the concept of a “hard shell” and “soft shell” structure within blocklets (Figure 2) [27]. The hard shell comprises regular blocklets, while the soft shell consists of defective blocklets. Alternating hard and soft shells give rise to the growth ring morphology characteristic of starch granules [28]. This ordered arrangement of crystalline structures contributes to the anisotropic optical properties of starch granules, known as birefringence. When observed under polarized light, starch granules exhibit a Maltese cross pattern, confirming their anisotropic structure and the symmetry of their crystalline organization [29].

## 3. Phase Transitions of Starch During Thermal Processing

### 3.1. Starch Gelatinization

Starch gelatinization is an irreversible phase transition process through which starch granules absorb water, swell, and form a gel-like substance upon heating [30,31]. This intricate process involves four distinct steps. (1) Water absorption and granule swelling: initially, water molecules permeate into the amorphous regions of starch granules, causing them to swell [32]. (2) Granule breakage: As the temperature rises and water infiltration continues, stress shifts to the crystalline regions, leading to their disintegration into an amorphous state. This transition eliminates birefringence and releases amylose into the surrounding solution [33]. (3) Molecular reorganization: the released starch molecules reorganize under the action of water, forming a network structure [21]. (4) Gel formation: upon cooling, intermolecular hydrogen bonds re-establish, stabilizing the gel structure [34]. Due to the complexity and heterogeneity of starch granules, gelatinization occurs across a temperature range, rather than at a single point, involving multiple endothermic events. The key thermodynamic parameters used to characterize this process include the gelatinization onset (To), peak (Tp), and conclusion (Tc) temperatures, as well as gelatinization enthalpy (ΔH) [35,36,37].

#### Factors Influencing Gelatinization

Starch gelatinization is influenced by intrinsic molecular structures and external environmental factors, both of which determine the energy required for granule disassembly. Key parameters such as To, Tp, and Tc temperatures, along with ΔH, are closely linked to factors like granule size, crystallinity, the amylose-to-amylopectin ratio, and chain length distribution [18,38,39,40,41]. Granule size and crystallinity: Larger granules with looser internal structures are more prone to water penetration, facilitating swelling and gelatinization. However, starches with higher crystallinity require greater energy and exhibit higher gelatinization temperatures and enthalpy because water initially penetrates the amorphous regions, transferring stress to the crystalline regions [18,38]. Amylopectin structure: Amylopectin consists of chains of different lengths, and the length distribution of these chains affects its ability to form double helices, which in turn affects the gelatinization temperature. Longer chains tend to form more stable double helices and require higher temperatures to gelatinize. The A (~DP 6–12) and B1 (~DP 13–24) chains of amylopectin form a double-helical structure in the semi-lattice of starch particles [42]. The thermal stability of these double helices is positively related to the gelatinization temperature of amylopectin [43]. And the trans-lamella chains (DP 37–69) forming long amylopectin double helices protruding from crystalline lamellae into continuing amorphous lamellae are decisive to Tc because of their higher thermal stability [33]. Amylose contribution: Amylose exhibits a more complex impact on gelatinization, extending the temperature range (Tc-To) through interactions with amylopectin helices. These interactions stabilize double helices in certain amylopectin segments, influencing ΔH in a nonlinear manner [44,45].

The water content, among external factors, significantly influences the order and rate of gelatinization [32,46]. With insufficient water content, the process of starch gelatinization may exhibit multi-stage heat absorption peaks, starches may need higher temperatures to start gelatinizing, and the crystalline structure may not be completely destroyed, resulting in incomplete gelatinization. The more moisture, the easier the starch granules are to fully absorb water expand and gelatinize faster [47].

Salt ions also significantly influence gelatinization, with their effects depending on the type and concentration of the salts involved. Salts affect gelatinization through three mechanisms: structural disruption or formation, electrostatic interactions, and competition for water molecules. Structurally, salts alter the hydrogen bonding network of water, thereby influencing starch–water interactions. Electrostatic interactions occur when charged ions interfere with hydroxyl groups on starch molecules, disrupting hydrogen bond formation. Lastly, salts compete with starch for water molecules, impacting hydration and the gelatinization process [32,48,49,50]. For instance, magnesium chloride promotes hydration and enhances gelatinization, while high-charge-density ions like sulfate (SO_4_^2−^) and calcium (Ca^2+^) increase the gelatinization temperature by forming structured water. In contrast, low-charge-density ions such as iodide (I^−^) and thiocyanate (SCN^−^) disrupt water structure, lowering the gelatinization temperature [51]. Sodium chloride exhibits concentration-dependent effects, increasing To at low concentrations (<2 mol/L) but decreasing it at higher levels due to changes in ion–starch interactions [52,53].

Lipids and proteins also modulate starch gelatinization. Lipids, including long-chain fatty acids, monoglycerides, and phospholipids, form complexes with amylose, stabilizing its helical structure and increasing the gelatinization temperature. These lipids compete with water molecules for binding sites within starch granules, reducing water absorption and limiting granule swelling during heating. Additionally, lipid molecules may create a barrier on the granule surface, further restricting water penetration [54,55,56,57]. Proteins surrounding starch granules act as a “defensive shell,” raising gelatinization temperature by delaying water access. In combination with lipids, proteins form ternary complexes with higher thermal stability and enthalpy [58,59,60,61].

Sugars and sugar alcohols influence starch gelatinization by stabilizing crystal structures, forming hydrogen bonds with starch molecules, and altering water activity. Their stereochemical properties, such as molecular size, flexibility, and hydroxyl group arrangement, determine their effectiveness. Sugars with more equatorial and exocyclic hydroxyl groups are particularly adept at enhancing crystal stability, increasing the gelatinization temperature and enthalpy [62,63,64,65,66,67].

Non-starch polysaccharides regulate gelatinization differently, as their large molecular size prevents them from infiltrating starch granules. Instead, they form a physical barrier around the granules, limiting water penetration and swelling. These polysaccharides also significantly impact solution viscosity. Their effects vary based on the type, concentration, and interaction with starch. For example, guar gum and xanthan gum reduce the gelatinization temperature and swelling force, while carboxymethyl cellulose has the opposite effect [68,69,70,71].

### 3.2. Starch Retrogradation

Starch retrogradation, also known as starch aging, refers to the process in which the molecular chains of gelatinized starch reorganize through hydrogen bonds into an ordered structure during cooling under certain conditions [72,73,74]. The retrogradation involves the transition of gelatinized starch molecules from a state of disorder to one of order. During the process of gelatinization, starch molecules undergo a transition from an ordered or partially ordered state to a high-energy disordered state under the influence of external energy, such as heat or pressure [10]. Upon cooling, starch molecules undergo intermolecular and spatial conformation rearrangement with water molecules, leading to their transformation into a low-energy ordered state. As temperature decreases during recirculation, molecular motion diminishes, and the side chains of amylose and amylopectin molecules tend to realign in parallel orientation through hydrogen bonding [75]. This leads to their closer proximity and the reformation of microcrystalline bundles, ultimately resulting in precipitation or gel formation. Based on the kinetic properties of starch retrogradation recrystallization, the process can be divided into four stages: conformational change of starch chains, the formation of double helices, crystal nucleus induction and crystallization growth, and perfect crystallization formation [76,77]. Depending on the duration of retrogradation and the state of molecular motion, the process of starch retrogradation can be classified into short-term retrogradation and long-term retrogradation. Short-term retrogradation typically occurs within a few hours to 24 h after starch gelatinization and is primarily dominated by amylose due to its smaller molecular weight and fewer side chains [78]. The directional movement of amylose chains facilitates intermolecular hydrogen bonding, resulting in the formation of a double helices structure and gradually progressing towards the development of a three-dimensional network structure. Then, with the extension of time, amylopectin with larger molecular chains and more side chains forms crystalline clusters through intermolecular hydrogen bonding, which intertwine with each other and lead to long-term at a much slower rate, typically requiring several weeks [77,79,80].

Retrogradation can be considered the inverse process of gelatinization; thus, the factors influencing gelatinization also impact retrogradation. Similarly, in terms of intrinsic factors, there is a positive correlation between amylose content and retrogradation rate. Amylose forms double-helical structures through intermolecular hydrogen bonding, which serve as connecting points during the retrogradation process [81]. A higher amylose content leads to increased aggregation of molecules through hydrogen bonding, resulting in a higher enthalpy during starch regeneration and a faster rate of regeneration [80,82]. The recovery of amylose is influenced by the length of its molecular chain, with longer chains experiencing steric hindrance that impedes orderly arrangement and retards regeneration, while shorter chains are more readily dissolved and pose challenges for revival [19]. It has been observed that amylose with a polymerization degree ranging from 250 to 1100 can rapidly regenerate within 100 min [19,83]. For long-term retrogradation, the length and distribution of amylopectin’s side chain play a crucial role in the process. The side chain of amylopectin must consist of at least 10 glucose units to enable the formation of a crystalline structure through a double helix arrangement. Conversely, shorter side chains with fewer than 10 glucose units impede retrogradation [79,84,85]. Additionally, the proportion of short side chains with a polymerization degree (DP) ranging from 9 to 11 in amylopectin significantly impacts the starch recovery rate. Slower starch retrogradation is observed with a decrease in the proportion of short side chains [85,86]. The presence of amylose facilitates the retrogradation of amylopectin, and an increase in amylose content leads to faster long-term retrogradation [87,88]. A synergistic effect between amylose and amylopectin retrogradation is evident, where the short-term retrogradation of amylose acts as a crystal nucleus for the recrystallization of amylopectin, promoting its retrogradation. However, it should be noted that, while amylose promotes the recrystallization and accelerated retrogradation of amylopectin, it does not impact its final crystallinity [72,89].

The retrogradation of amylopectin is closely associated with the water content within the system. Amylopectin retrogradation primarily involves B-type crystallization, which requires a higher water content compared to A-type crystallization. Water serves two main functions in amylopectin regeneration: firstly, as a plasticizer, it facilitates the migration and orderly arrangement of amylopectin molecules; secondly, water actively participates in the recrystallization process by acting as bound water [32]. The promotion of amylopectin recrystallization via water is dependent on its content. It has been observed that wheat starch recrystallization increases with increasing water content within the range of 27% to 50%, and recrystallization decreases with a further increase in water content [90]. Temperature is another decisive factor. When the temperature of starch paste surpasses its melting temperature (Tm), molecular movement becomes intense, resulting in a disordered distribution of amylopectin molecules that cannot be recrystallized effectively. On the other hand, if the temperature falls below the glass transition temperature (Tg), molecular chain movement freezes, leading to limited directional migration over a short period of time. However, when the retrogradation temperature ranges between Tg and Tm, a thermodynamically unstable state occurs, allowing significant directional migration for achieving ordered arrangement through hydrogen bonding interactions and ultimately leading to the attainment of thermodynamic stability and a subsequent reformation into crystals [84,91,92].

**Figure 3:**
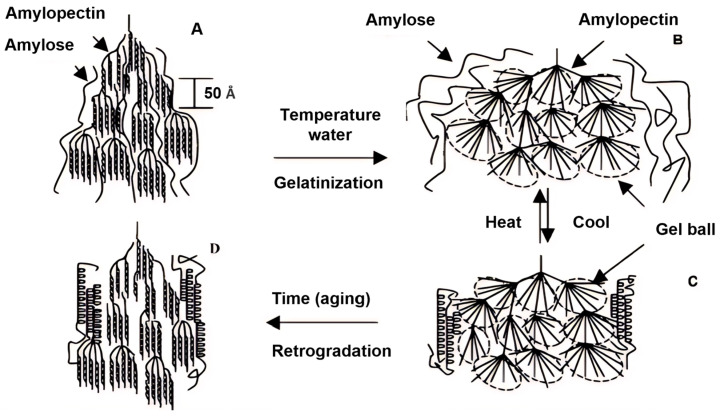
Schematic representation of the phase transitions of starch during thermal processing and aging [94]. (**A**) native starch structure; (**B**) gelatinization; (**C**) cooling and gel formation and (**D**) retrogradation.

Figure 3 provides a schematic representation of the retrogradation process following starch gelatinization. During gelatinization, the double-helical crystalline structures in amylopectin are disrupted, breaking apart the crystalline regions. However, the amylopectin chains retain a degree of regularity and reorganize into gel-like aggregates, often referred to as “gel-balls.” These gel-balls primarily consist of chains originating from the same sub-main chain of amylopectin. The molecular entanglements between gel-balls and larger aggregates, such as super-globules, are significantly fewer compared to those between linear polymer chains. This is due to the relatively short length and compact arrangement of the amylopectin chains within gel-balls. During the recrystallization or retrogradation process, amylose initially forms V-type single-helical crystals. Meanwhile, gelatinized amylopectin remains amorphous immediately after gelatinization. Over time, however, the crystallinity of amylopectin gradually increases, contributing to the structural changes associated with retrogradation [93].

The presence of cations not only decelerates the rate of retrogradation but also hinders the recrystallization rate [95]. Divalent cations (such as Ca^2+^ and Mg^2+^) have a more obvious effect in comparison to monovalent cations (such as Li^+^, NH^4+^, Na^+^, and K^+^) [96,97]. This phenomenon is believed to be associated with the higher charge density of divalent cations, resulting in stronger hydration and subsequently lower water activity. Ciesielski and Tomasik argued that the hydrogen bond between starch molecules and the complex association between starch and metal cations were in competition with each other, so the complex association between cations and starch in the presence of salt might be a reason for the reduction in starch retrogradation (dependent on the intermolecular hydrogen bond) [98].

The additives, including lipids, carbohydrates, and proteins, impact the retrogradation at different stages via various mechanisms [76,99]. Lipids and carbohydrates (monosaccharides polysaccharides, oligosaccharides, and polyols) are generally suppressive for starch retrogradation. Lipids constrain the starch molecules’ movement by forming a complex with starch and physically hindering the proximity and rearrangement of molecule chains [57]. Lipids may also affect the dynamics of starch chains and reduce the fluidity of starch chains [68]. It is worth noting that instances in which carbohydrates promote retrogradation are relatively rare and require specific conditions or concentrations. Most carbohydrates inhibit retrogradation by competing with starch molecules for water, thereby forming physical barriers or altering the microscopic structure of starch gels [99,100]. Moreover, retrogradation is a complex process influenced by various factors, such as the starch type, water content, temperature, pH value, and other components within the system. Therefore, the specific effects of carbohydrates on retrogradation may vary, depending on experimental conditions and food systems. Proteins display a multiple and concentration-dependent influence on retrogradation. If the protein content is too high, it may form disulfide bonds, which may restrict water migration, thereby reducing its anti-retrogradation ability [101]. And in different retrogradation stages, proteins may have different effects, which may inhibit retrogradation in the early stage and promote it in the long term [101]. Rice protein and glutenin in wheat have been reported to delay retrogradation, while starch granule-associated proteins (SGAPs) accelerate the retrogradation process by competitively binding with water molecules and thus enhancing the hydrogen-binding interactions between starch molecules [102].

### 3.3. Phase Transition Under Shearless and Shear Strength Conditions

Starch undergoes phase transitions from a semi-crystalline to an amorphous structure due to factors such as heat, moisture, and shear force. Upon cooling gelatinized starch, the molecules reorganize through the re-establishment of hydrogen bonds, resulting in processes like retrogradation or recrystallization. These transformations include granule expansion, dissolution, and gelatinization, with external energy input playing a significant role in initiating these changes [103,104,105]. Shear force is particularly influential, not only facilitating the expansion and disintegration of starch granules but also affecting the alignment of starch molecules. This has practical implications for food processing and material preparation. During gelatinization, under shearless conditions, starch granules require sufficient water (over 70%) and proper temperature for swelling and eventual collapse. Conversely, in the presence of shear force, the process requires less water. Shear disrupts hydrogen bonds within and between molecules, distorting the double-helical structure of starch and accelerating water molecule binding to free hydroxyl groups, thereby expediting gelatinization [106,107,108].

Starch is not inherently suitable for use as a synthetic plastic substitute due to its higher glass transition and melting temperatures compared to its decomposition temperature (225–250 °C) [91]. However, the production of thermoplastic starch is achievable through approaches that generate sufficient shear force. Shear disrupts molecular bonds, reducing the reliance on water penetration to dissolve crystals. Common methods for thermoplastic starch synthesis include single- and twin-screw extrusion, kneading, casting, and pressing, with extrusion and casting being the most widely employed [109,110,111]. The extrusion process is particularly influenced by operating conditions such as the screw rotation speed (SRS), temperature, and moisture content. SRS directly affects the residence time of materials in the extruder, the degree of mixing, and the thermal treatment of starch-based materials. Seligra et al. demonstrated that, at a low SRS (40 rpm), insufficient mechanical energy results in the incomplete disintegration of starch granules, leaving ungelatinized particles that can act as sites for crack propagation and impair uniformity and functionality. At a moderate SRS (80 rpm), starch particles break down effectively, increasing the contact area between starch and plasticizers, which enhances interfacial interactions and improves film toughness. At a high SRS (120 rpm), excessive shear force may over-disrupt starch particles, weakening interfacial interactions and reducing toughness. The co-extrusion of other components such as plasticizers, polymers, or nanofillers with starch further underscores the critical role of SRS in achieving uniform mixing and optimal interfacial interactions [112,113,114]. Moisture content also plays a critical role during extrusion. Insufficient moisture results in weak inter-granular bonding, reducing film tensile strength and elongation at break. Adequate moisture promotes gelatinization and plasticization, improving film flexibility and extensibility. However, excessive moisture may create structural inconsistencies or bubble formation, compromising mechanical properties during drying.

Additionally, shear force significantly impacts the rheological properties of starch during thermal processing. Using a rheometer, Yu and Chen observed that corn starch granules maintained structural stability through a balance of structural degradation and continuous gelatinization under a constant shear rate (5 s^−1^) [105,115]. Viscosity increased steadily during gelatinization, peaking at 110 Pa·s between 70 and 75 °C before declining due to starch-structure degradation. This progression highlights the breakdown of starch granules and the phase separation of amylose and amylopectin. The complete breakdown of starch granules under continuous shear results in shear thinning, a common rheological phenomenon.

Shear force also plays a crucial role in modulating starch retrogradation, which is essential for maintaining product quality. Properly applied shear enhances thermal stability by promoting compact and uniform gel networks. Zeng et al. found that extrusion-induced molecular alterations in chestnut starch accelerated short-term recrystallization (1 day) but impeded long-term ordered crystallization (21 days) due to chain damage and reorganization [116]. Further studies by Zhu et al. showed that shear force and high-temperature exposure fragmented starch molecular chains, particularly the elongated segments of amylopectin. This fragmentation led to the conversion of branched amylopectin into more linear amylose, promoting short-range ordered structures while inhibiting long-range crystallization. Consequently, retrogradation kinetics were decelerated, improving gel stability and quality [117].

## 4. Improving Toughness via Plasticizers

Plasticizers play a crucial role in starch-based materials’ production and processing, improving flexibility and workability by modifying the mechanical properties of starch. The plasticization mechanism involves plasticizers penetrating into starch molecules, weakening the intermolecular forces between starch chains [8,118]. The primary mechanism involves the interaction between polar plasticizer groups and hydroxyl groups on starch, displacing hydrogen bonds among starch molecules. Concurrently, non-polar plasticizer groups isolate and shield the hydroxyl groups, thereby reducing van der Waals forces between starch molecules. This results in the increased mobility of starch chains, leading to enhanced material flexibility [119,120].

### 4.1. Water

Water is one of the most widely used plasticizers in starch-based materials, serving as both a plasticizer and gelatinization agent. By disrupting hydrogen bonds, water transforms starch from a rigid to a flexible state. However, its low boiling point leads to volatilization during processing and storage, causing the material to become brittle over time [63,118,121,122,123]. This limitation underscores the need for more stable plasticizers.

### 4.2. Polyols and Saccharides

Polyols, such as glycerol, sorbitol, and xylitol, are widely used as plasticizers in starch-based materials due to their excellent compatibility with starch, which is primarily attributed to their multiple hydroxyl (-OH) groups. The plasticization efficiency of polyols is closely related to the number and arrangement of these hydroxyl groups. Polyols with more hydroxyl groups interact more effectively with starch molecules by forming hydrogen bonds, which reduce intermolecular forces and increase chain mobility. Conversely, polyols with fewer hydroxyl groups or longer alkyl chains, such as pentanol or hexanol, exhibit poor compatibility and lower plasticization efficiency due to limited interaction with starch. Short-chain di-alcohols, such as ethylene glycol, display enhanced compatibility because their shorter -R chains minimize steric hindrance, allowing better integration with the starch matrix. Among polyols, those with a high density of hydroxyl groups, such as glycerol and sorbitol, are particularly effective in improving the flexibility and workability of starch-based materials [124,125,126].

Saccharides, including monosaccharides (e.g., glucose and fructose), disaccharides (e.g., sucrose and maltose), and dextrins, also play a significant role as co-plasticizers in starch-based systems. Their structural similarity to starch enables them to integrate seamlessly into the starch matrix. Linear saccharides, such as fructose and xylose, are especially effective in disrupting the ordered crystalline regions of starch, thereby enhancing polymer chain mobility and plasticization efficiency. By contrast, cyclic saccharides, such as cyclodextrins, tend to stably incorporate into the starch matrix without significantly altering its microstructure, making them suitable for applications where structural integrity must be preserved. The use of saccharides as co-plasticizers with water or polyols has shown synergistic effects, improving the overall plasticization efficiency. For instance, the combination of polyols and saccharides can enhance hydrogen bonding networks, reduce the crystallinity of starch, and increase material flexibility. This synergy is particularly advantageous in applications requiring tailored mechanical properties and moisture resistance. Developing such synergistic plasticizing systems represents a promising direction for advancing the functionality and versatility of starch-based materials [122,127].

### 4.3. Other Polar Substances

In addition to polyols and saccharides, various other polar compounds, including amines, amides, carboxylic acids, their salts, and amino acids, have been extensively explored as effective plasticizers for starch. These substances typically exhibit higher boiling points and contain polar functional groups such as -COOH and -NH2, which form hydrogen bonds with hydroxyl groups on starch molecules. This interaction disrupts the native intermolecular and intramolecular associations within starch, thereby enhancing chain mobility and improving the flexibility and plasticization of starch-based materials [128,129].

Carboxylic acids, such as citric acid, succinic acid, oxalic acid, and adipic acid, are among the most widely studied plasticizers in this category. Citric acid has been identified as both a plasticizer and a chemical modifier during the melt processing of starch, as reported by Carvalho et al. [130]. Despite its efficacy in reducing crystallinity and enhancing flexibility, citric acid’s low molecular weight makes it prone to inducing water sensitivity and recrystallization over time, leading to unstable mechanical properties during storage and aging. Comparative studies on other acids have revealed that succinic acid interacts more effectively with starch than oxalic or adipic acid, yielding films with reduced crystallinity and improved thermal stability. The superior reactivity of succinic acid is attributed to its shorter chain length, which enhances its compatibility and interaction with the starch matrix, as evidenced by the formation of ester bonds that strengthen the material’s structural integrity [129].

Beyond carboxylic acids, other polar plasticizers, such as amines and amides, have demonstrated unique plasticizing effects. Studies comparing formamide, urea, glycerol, and ethylene glycol (EG) have highlighted the importance of functional groups in determining plasticization performance [128,131]. Among these, formamide exhibits the strongest hydrogen bonding interaction (H-O…H-N) with starch, resulting in exceptional plasticizing efficiency. Chen et al. [132] further demonstrated that hydroxyl groups in EG and amino groups in ethylenediamine (EDA) effectively form hydrogen bonds with starch chains, significantly enhancing flexibility and processability.

Ethylbutylformamide (EBF) offers a more complex interaction with starch. Its amide groups establish hydrogen bonds not only with hydroxyl groups but also with ether bonds along the starch backbone. This dual interaction mechanism contributes to superior plasticization efficacy, as it simultaneously disrupts crystalline regions and enhances amorphous chain mobility.

The diversity of polar plasticizers, combined with their varied functional groups and molecular interactions, underscores their potential in tailoring starch-based materials for specific applications. The continued exploration of these compounds, particularly in synergistic systems or modified forms, holds promise for further advancing the performance and versatility of starch-based bioplastics.

### 4.4. Novel Plasticizers

Recent advancements in starch plasticization have introduced ionic liquids (ILs) and deep eutectic solvents (DESs) as promising novel plasticizers, offering unique advantages over traditional options [133]. ILs, which are entirely composed of organic salts, exhibit exceptional properties, including non-volatility, non-flammability, and excellent thermal and electrochemical stability. Imidazolium-based ILs, such as 1-butyl-3-methylimidazolium chloride ([BMIM]Cl) and 1-ethyl-3-methylimidazolium acetate ([Emim]Ac), have demonstrated remarkable plasticizing effects on starch, particularly when used in combination with conventional plasticizers like glycerol [134,135]. The synergistic use of ILs and glycerol significantly reduces starch crystallinity, water content, and the glass transition temperature, resulting in enhanced flexibility and processability.

Similarly, DESs and low-transition-temperature mixtures (LTTMs) have emerged as effective alternatives for starch plasticization. DESs, formed from mixtures of hydrogen bond donors and acceptors, possess lower phase transition temperatures than their individual components. This property allows them to effectively disrupt the ordered crystalline regions of starch and promote the formation of more amorphous structures. DESs are also less hygroscopic than traditional plasticizers, further enhancing the stability and moisture resistance of starch-based materials [136,137].

The blending of multiple plasticizers has proven to be a highly effective strategy for enhancing starch plasticization. Studies have demonstrated that combining glycerol with sorbitol, urea with ethanolamine, or urea with formamide results in stronger molecular interactions with starch compared to the use of individual plasticizers. Mixtures of sugars with glycerol have similarly shown improved plasticization efficiency, attributed to their complementary hydrogen bonding and disruption of crystalline regions [137,138]. The exploration of ILs and DESs, along with their synergistic combinations with traditional plasticizers, represents an exciting frontier in starch plasticization. Future research should focus on understanding the long-term stability, biodegradability, and potential environmental impacts of these novel plasticizers. Additionally, the development of bio-based ILs and DES could further align starch-based materials with the goals of sustainability and environmental friendliness.

## 5. Chemical Modifications

**Figure 4:**
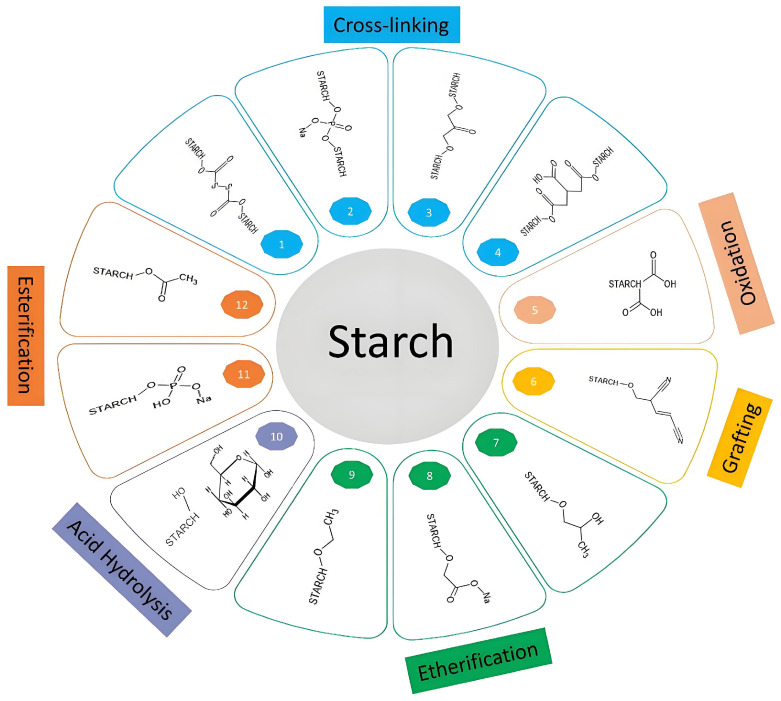
Chemical modification on starch. Cross-linking modification with (**1**) CS_2_, (**2**) POCl_3_, Na_3_P_3_O_9_, (**3**) epichlorohydrin, and (**4**) C_6_H_5_O(COOH)_3_; oxidation modification with (**5**) O_3_ and HIO_4_; grafting modification with (**6**) acrylamide with possible initiators; etherification modification with (**7**) C_3_H_6_O, (**8**) CH_2_ClCOONa, and (**9**) C_2_H_5_Cl; acid hydrolysis with (**10**) HCl, TFA, and HNO_3_; esterification modification with (**11**) Na_2_HPO_4_ and (**12**) CH_3_COOH [139].

The abundant hydroxyl groups in starch provide versatile sites for a wide range of chemical modifications, enabling significant alterations to its properties. By introducing active functional groups, it is possible to modify key characteristics of starch-based materials, such as aqueous solubility, gelatinization temperature, and hydrophobicity, alongside structural and crystallinity changes. Common chemical modification methods include esterification, acetylation, etherification, oxidation, polymer grafting, cross-linking, and hydrolysis (Figure 4) [94,139]. The extent of starch modification is typically measured using two parameters: the degree of substitution (DS) and molar substitution (MS). DS reflects the average number of hydroxyl groups on the starch glucose unit that have been replaced with substituents, with a theoretical maximum of 3.0, corresponding to the three hydroxyl groups available per glucose unit. In contrast, MS represents the average number of substituents attached to either the hydroxyl groups on the starch glucose unit or pre-existing substituents. Unlike DS, MS can exceed 3.0 due to the addition of substituents to previously introduced groups [140,141]. This versatility in modification provides a powerful approach to tailoring starch’s functionality in order to meet the demands of diverse applications, from improving water resistance in packaging materials to enhancing the thermal stability of starch-based composites.

### 5.1. Esterification

Esterification is a common modification method for the preparation of hydrophobic and thermoplastic starch. Due to the presence of a multitude of hydroxyl groups on starch molecules, they can form esters with various organic or inorganic acids, resulting in the formation of starch derivatives with lipophilic groups. The esterifying agents commonly employed include organic carboxylic acids (acetic acid, propionic acid, butyric acid, and others), acid chlorides (including acetyl chloride and vinyl chloride acids), anhydrides (acetic anhydride, propionic anhydride, and octenyl succinic anhydride), inorganic acids (phosphoric acid, nitric acid, and sulfuric acid), enol esters (vinylacetic acid and allyl acetic acid), and polyhydric alcohols (glycerol, sorbitol, and mannitol) [142,143,144,145].

The implementation of esterification has demonstrated various advantages in terms of starch film production. The inherent polyhydroxyl nature of starch imparts excellent hydrophilicity, but this characteristic, along with its inferior mechanical properties, limits its application potential, particularly in wet environments. Esterification serves as an effective strategy to overcome these limitations [146,147]. Esterified starch not only exhibits a reduced degree of retrogradation and diminished occurrences of paste gelation and dehydration condensation phenomena but also undergoes alterations in sugar permeability, glossiness, viscosity characteristics, gel texture formation ability, film-forming capacity, thermal stability, and emulsification stability within the starch paste matrix. The formation of starch esterified occurs through the reaction between the hydroxyl group in the starch molecule and either an inorganic acid or a carboxylic acid derivative, resulting in a weakening of intermolecular hydrogen bonds [148]. As a result, esterified starch demonstrates heightened levels of hydrophobicity and thermoplasticity compared to its original form. Furthermore, this process significantly enhances both mechanical strength and toughness, making esterified starch suitable for extensive applications across diverse industries such as food processing and preservation, medicine production processes, textile manufacturing industry, composite materials’ development, paper production, and the environmental protection field [148,149,150,151].

It is worth noting that the type and length of the carbon chain in these agents significantly influence reaction kinetics. The reactivity of a fatty acid typically increases as its chain length decreases. Short-chain fatty acids, such as acetyl chloride and propionyl chloride, exhibit a higher likelihood of reacting with starch molecules due to their enhanced accessibility and penetration into starch granules. Consequently, this facilitates achieving a higher degree of substitution. Fatty acids of varying chain lengths can lead to distinct substitution patterns. For instance, studies have suggested that the utilization of short-chain fatty acid derivatives (such as acetyl chloride) is more likely to result in substitution reactions occurring at positions C_2_ and C_3_ on the starch molecule, whereas long-chain fatty acid derivatives may promote greater substitution at position C_6_ [152]. Correspondingly, the crystallinity, toughness, strength, and hydrophobicity of starch films vary. Short-chain fatty acid derivatives, such as acetic acid and propionic acid, typically enhance the water solubility and absorption of starch by increasing its hydrophilicity. On the other hand, long-chain fatty acid derivatives like palmitic acid and stearic acid augment the hydrophobicity of starch, potentially reducing its water solubility but enhancing its solubility in non-polar solvents. Long-chain fatty acid-modified starches often exhibit improved thermal stability and mechanical properties, making them advantageous for high-temperature processing and various applications [153,154,155].

### 5.2. Etherification

Etherified starch is a derivative of native starch in which its active hydroxyl groups are chemically modified to form ether bonds with etherifying agents via oxygen atoms. Common etherification agents include epoxides (e.g., epoxypropane, ethylene oxide, and 2,3-epoxypropyltrimethylammonium chloride), acrylic acid derivatives (e.g., methyl methacrylate, methacrylic acid, and 2-ethylhexyl acrylate), alkyl chlorides (e.g., butyl chloride, octyl chloride, dodecyl chloride, and benzyl chloride), maleates (e.g., diethyl maleate, dipropyl maleate, and dibutyl maleate), dimethyl sulfuric acid, and halogenated hydrocarbons containing iodine or bromine [21,148,156,157].

Etherification typically occurs in an alkaline aqueous medium. In this environment, starch hydroxyl groups are converted into alkoxide anions, which readily react with etherifying agents. Several factors influence the efficiency of the reaction, including pH, temperature, reaction time, starch granule properties (e.g., particle size, shape, porosity, crystallinity, and surface characteristics), the concentration of etherifying agents, pretreatment techniques, and operating parameters such as stirring speed and pressure [158,159,160]. Innovative approaches have been developed to improve etherification efficiency and introduce new functionalities. For instance, Xie et al. utilized a reactive extrusion process under alkaline conditions to prepare carboxymethyl starch (CMS) with amphiphilic properties by reacting CMS with cetyl bromide (CB). This method demonstrated significantly higher hydrophobic modification efficiency compared to traditional batch processes, highlighting the potential of extrusion technology in modifying starch properties [161].

Starch ethers are categorized as ionic or nonionic, based on their charge characteristics in aqueous solutions. Ionic starch ethers: examples include carboxymethyl starch and quaternized starch. Carboxymethyl starch, with its negatively charged groups, improves water solubility and enhances repulsion between starch molecules, leading to better hydration and swelling properties. Quaternized starch, modified with cationic quaternary ammonium groups, imparts a positively charged surface, improves water absorption and solubility, and enhances biodegradability. These ionic modifications enable starch ethers to function as polymer electrolytes, expanding their potential applications in bio-based films, adhesives, and coatings [161,162,163]. Nonionic starch ethers: hydroxyethyl starch (HES) and hydroxypropyl starch (HPS) are prominent nonionic derivatives. The introduction of larger hydrophilic side groups reduces intermolecular interactions, inhibits retrogradation, and stabilizes viscosity. These modifications also enhance the mechanical properties of starch films by decreasing hardness and brittleness, improving transparency, and increasing flexibility [157,164].

Hydrophobic side groups can also be introduced onto starch via etherification to create thermosensitive or water-resistant properties. For example, Jong et al. synthesized a hydrophobic thermosensitive polymer, 2-hydroxy-3-(2-propynyloxy)propyl hydroxyethyl starch (PyHES), by modifying hydroxyethyl starch with 2-propylglycidyl ether (PGE) in an aqueous medium. Such modifications yield versatile starch derivatives suitable for applications requiring tailored water resistance, thermal sensitivity, or compatibility with hydrophobic matrices [165,166]. The etherification of starch plays a pivotal role in advancing starch-based films. The reduced intermolecular forces and enhanced compatibility achieved through etherification improve film flexibility and transparency while minimizing retrogradation and brittleness. Additionally, amphiphilic starch ethers facilitate uniform dispersion in blends with hydrophobic components, broadening their utility in creating high-performance biodegradable packaging materials. Ionic starch derivatives can further enhance the moisture resistance and mechanical strength of starch films, making them competitive alternatives to petroleum-based plastics.

### 5.3. Oxidization and Acid Hydrolysis

The oxidation and acid hydrolysis of starch can both introduce carbonyl and/or carboxyl groups onto the starch molecule. However, the oxidation process involves the initial conversion of the hemiacetal group at C1 of the glucose ring into a carboxyl group, followed by successive oxidations of the C6 aldehyde group to a ketone and then to a carboxylic acid group. Additionally, the vicinal hydroxyl groups at C2, C3, and C4 are oxidized to carbonyl groups, which further undergo oxidation to form carboxyl groups. This oxidative transformation also leads to the breaking of glycosidic bonds and the weakening of glycosidic linkages. Ultimately, these reactions result in degradation of starch molecules [167,168,169]. Common oxidizing agents include sodium hypochlorite, hydrogen peroxide, ozone, sodium periodate, and sodium permanganate. Novel oxidation methods, such as photocatalytic oxidation, enzymatic oxidation, electrochemical oxidation, and pulsed electric field (PEF) treatments, have been developed to improve efficiency and reduce environmental impacts [3,4,142].

Oxidation increases the hydrophilicity of starch by converting hydroxyl groups into carbonyl and carboxyl groups, enhancing hydrogen bonding between molecules. This modification can improve the inner cohesion and mechanical strength of starch films. For example, oxidized cassava starch films exhibit increased tensile strength and Young’s modulus but decreased ductility, making the films more brittle. This indicates that enhanced hydrogen bonding, while improving rigidity, reduces flexibility [170,171]. Additionally, the impact of oxidation on the crystallinity of starch can influence its mechanical properties. Optimizing parameters such as the degree of oxidation, starch origin, and film preparation processes can balance mechanical strength with ductility and toughness, tailoring films for specific applications.

The acid hydrolysis of starch primarily involves the cleavage of α-1,4 and α-1,6 glycosidic bonds, resulting in chain fragmentation and the formation of new reducing ends. Acids first target the amorphous regions of starch granules, where molecular interactions are weaker, producing smaller molecular fragments. This process alters the molecular structure of starch, with significant effects on its mechanical and thermal properties [158,172,173]. The degree of hydrolysis, as well as the type and concentration of acid, reaction time, temperature, and starch source, determine the extent of structural changes. Moderate hydrolysis can break down molecular chains, reducing intermolecular interactions and consequently lowering tensile strength and modulus of elasticity. However, this reduction in rigidity can also increase elongation at break, making starch films more flexible and less brittle [170,174]. Common acids used for hydrolysis include hydrochloric acid, sulfuric acid, and phosphoric acid. Citric acid, a food-grade organic acid, is often employed for gentle hydrolysis, particularly in applications where the properties of citric acid are desired in the final product. Citric acid’s dual role as a hydrolyzing agent and functional additive makes it suitable for biodegradable food packaging and other environmentally friendly applications [175].

Both oxidation and acid hydrolysis significantly influence the mechanical properties of starch films, such as tensile strength, elongation, and rigidity. Oxidation generally increases stiffness and mechanical strength but may reduce flexibility, whereas acid hydrolysis can increase flexibility at the cost of reduced strength. Careful control over treatment conditions allows for the customization of starch films to meet specific requirements, such as improved water resistance, tailored mechanical properties, or enhanced compatibility with other polymers. Future research should focus on optimizing these processes to minimize environmental impact while maximizing the functional properties of starch-based materials for applications in sustainable packaging, biomedical devices, and advanced composites.

### 5.4. Grafting

Grafting modification is a chemical method that are able to enhance the physical, chemical, and mechanical properties of starch by grafting monomers (such as methyl methacrylate, acrylic acid, butyl acrylate, lactide) onto the molecular chain of starch through a copolymerization reaction [176]. This technique facilitates improved thermoplasticity, enhanced mechanical strength, superior water resistance, and reduced moisture absorption in starch, thereby broadening its applications to diverse fields, including packaging materials, sustained-release drug carriers, paper processing, and biomaterials [177,178]. The grafting method could be classified as “grafting from” and “grafting onto” [179]. In the former method, monomer polymerization is initiated directly on the starch molecular chain. Starch molecular chains can serve as initiators due to the presence of active functional groups in their structure or through functional groups introduced after chemical modification. The formation of graft copolymers involves the polymerization of monomers onto starch molecules through either free radical or ring-opening polymerization [180]. A typical “grafting from” reaction involves the copolymerization of starch-grafted polyacrylic acid (starch-g-PAA). This type of grafting reaction is initiated via the decomposition of initiators, such as ammonium sulfate (APS), potassium persulfate (KPS), ammonium cerium nitrate (CAN), or Fenton’s initiator, to generate free radicals [180,181]. These radicals then react with the hydroxy groups on starch and subsequently react with monomers (such as acrylic acid, methyl methacrylate, and methacrylic acid (MAA)), thereby initiating the polymerization of monomers onto starch [177]. The properties of starch graft copolymers can be tailored by selecting different monomers, controlling the grafting ratio, and adjusting the grafting density, thereby enabling the precise regulation of the mechanical characteristics of films. The starch-g-PMAA films synthesized by Weerapoprasit and Prachayawarakorn exhibited a decrease in crystallinity, reduced mechanical strength, lower Young’s modulus (i.e., stiffness), and enhanced flexibility with an increase in the MAA grafting percentage [182].

The “grafting onto” method refers to the covalent attachment of a pre-synthesized polymer chain with a reactive end group onto starch chains through a chemical reaction. This requires the presence of functional groups on starch molecules capable of undergoing reactions with polymer end groups. Tai et al. prepared an ultra-flexible starch–polyurethane film by grafting PEG-iso onto starch to form an interpenetrating polymer network. In the starch–polyurethane composite material, PEG serves as the soft segment, providing excellent flexibility, while the polyurethane network acts as the hard segment, offering necessary strength and rigidity. The compatibility and synergistic effect between these segments enable the film to exhibit exceptional toughness [183,184].

### 5.5. Other Modification Methods

**Figure 5:**
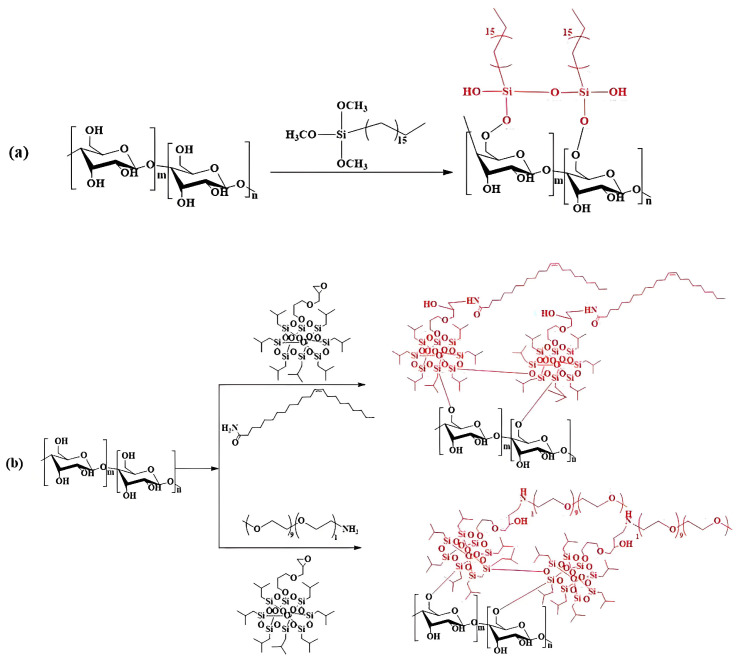
(**a**,**b**) Schematic summary of condensing reaction [142].

In the chemical modification of starch, condensing reaction refers to the introduction of new chemical groups onto starch molecules, leading to the formation of more intricate molecular structures. A typical example is the modification of starch using silane coupling agents (Figure 5) [142]. Silane coupling agents, such as hexamethyldisilazane (HMDS) or hexamethyldisiloxane (HMDSO), can form stable Si-O-C bonds with hydroxyl groups on starch molecules under acidic or alkaline conditions. Following silanization treatment, starch exhibits enhanced surface properties, heightened hydrophobicity, and improved compatibility with non-polar materials [185,186,187]. Additionally, silane coupling agents can react with the surfaces of starch molecules and nanofillers (such as SiO_2_) through their two distinct reactive groups, forming chemical bonds. The introduction of these chemical bonds aid in the formation of a cohesive and compact network structure, thereby enhancing the tensile strength and elongation at the break of the starch film. For instance, research has demonstrated that incorporating nano-SiO_2_ modified via silane coupling agents into starch–PVA composite films resulted in an increase in tensile strength ranging from 49.0% to 68.35% [188].

The chemical cross-linking of starch could create multiple networks within the polymer matrix and thus result in the enhancement of mechanical properties. Multifunctional acids and alcohols, dialdehyde, and epichlorohydrin are some commonly used cross-linking agents [189,190]. These agents generally react with the hydroxy groups as the generation of ether bonds or ester bonds. The cross-linking points could also be formed between starch and other polymers. Carboxymethyl cellulose (CMC) and starch were cross-linked by citric acid via esterification. Additionally, chemically modified starch could form novel cross-linking bonds. The cross-linked rice starch is prepared through the Schiff base reaction between oxidized rice starch and aminated rice starch. The resulting cross-linked starch films demonstrated exceptional toughness, as they possess a higher capacity for energy absorption prior to fracture. This enhanced toughness can be attributed to the formation of a more tightly interconnected network structure through cross-linking, which enables the effective dissipation and absorption of applied forces [191]. Notably, meticulous control of the degree of cross-linking is essential, as it directly impacts both the mechanical properties and processability of the starch films. If the degree of cross-linking is maintained at a moderate level, the film can obtain proper strength and good flexibility at the same time. However, excessive cross-linking can result in excessive rigidity and brittleness in the film, compromising its practicality; an increased degree of cross-linking may reduce the breaking elongation of the starch film due to constrained molecular chain mobility and reduced flexibility. This is attributed to the enhanced intermolecular interactions that restrict molecular chain sliding, thereby improving the film’s resistance to external forces. Furthermore, under external forces, an excessively cross-linked film is more prone to fracture, rather than deformation [192,193].

## 6. Composite with Other Hydrophilic Polymers

### 6.1. Starch–Cellulose Composite

Cellulose, due to its chemical similarity with starch, is the most commonly used polymer for enhancing starch film. This is because it facilitates interactions such as hydrogen bond formation, thereby increasing adhesive strength at the interface [194]. However, cellulose is indissoluble in traditional solvents such as water and most polar solutions due to complex non-covalent interactions within and between its molecules. Therefore, two methods can be employed for compositing cellulose with starch: (1). Transforming cellulose into aqueous soluble derivatives via chemical modification (e.g., carboxymethyl cellulose (CMC), methyl cellulose (MC), and hydroxypropyl methyl cellulose (HPMC)) [195,196,197]. These cellulose derivatives exhibit enhanced solubility, thereby demonstrating improved film-forming properties when composited with starch. Lan et al. employed a casting method to prepare a composite film embedded with Lactococcus lactis using corn starch (NS) and carboxymethyl cellulose (CMC) [198]. The composite film exhibited optimal mechanical properties at an NS–CMC ratio of 5:5, with a tensile strength of 4.62–5.83 MPa and an elongation at break of 78.59–86.75%. This edible and pliable composite film possesses excellent ductility, making it suitable for packaging nuts, biscuits, and candies. (2) Extracting nanocellulose (NC) from plant fibrils and incorporating it into a starch matrix, where the NC functions as a nano-filler or reinforcement building block [199,200,201]. The mechanical properties of the composite films are governed by various factors such as the aspect ratio and crystallinity of NC, processing conditions, the dispersion state, and compatibility between the NC and the starch matrix. According to the diameter and length of NC, it could be classified as nano-fibrillated cellulose (NFC), cellulose nanowhisker (CNW), cellulose nanocrystals (CNC), and bacterial cellulose (BC). Other special forms of nanocellulose, like spherical nanocellulose (SNC) and cellulose nanosheet (CNS), have been prepared and composited with starch as well [202,203,204].

As a high modulus and stiffness additive, through its incorporation, NC can significantly enhance the tensile strength, Young’s modulus, and puncture strength of starch films [205]. The reinforcement effect of NC generally improves with a higher aspect ratio (length-to-diameter ratio), as the bridging and stress dispersion capabilities of high-aspect ratio NC in composite materials enhance the mechanical strength of the material. High-aspect ratio NC is more likely to form a three-dimensional interlaced network structure in composite materials, which effectively restricts matrix deformation and enhances material stiffness and strength. Generally, longer fibers with higher aspect ratios exhibit better load transfer efficiency in the matrix due to their increased interaction with the matrix [199,206,207]. The NC content also plays a critical role in determining the mechanical properties of the composite film, as there exists an optimal concentration at which the mechanical performance reaches its peak. At this specific content level, NC is uniformly dispersed within the matrix, forming an efficient stress transfer network that maximizes the material’s mechanical properties. However, exceeding a certain threshold of NC content may lead to a decline in the composite’s mechanical properties. This could be attributed to excessive aggregation of NC, inadequate compatibility between the reinforcing agents and starch matrix, or compromised processing performance resulting from high levels of NC [199,208,209].

Methods for fabricating NC–starch composite films include solvent casting, hot pressing, blowing, extrusion, and other techniques with varying processing conditions [210,211,212]. Solvent casting is the most commonly used method, as it is more convenient in the preparation of multi-layer composite films, and this method can achieve good adhesion and uniform compounding between the layers of materials [213]. The novel layered structure of cellulose nanowhiskers (CNWs) in the fractured cross-section of the starch–CNWs composite film was observed by Liu et al. for the first time [214]. This achievement was accomplished through the precise control of the water evaporation rate, which exerted a significant influence on the dispersibility of CNWs during the film formation process. Slower evaporation allowed for increased self-rearrangement and arrangement time for CNWs, resulting in the formation of a layered structure within the starch matrix. But this method should ensure the sufficient dispersion of NC. The extrusion method can achieve the mixing and chemical reaction of materials in a single processing step, saving time and labor and improving production efficiency. Fourati et al. successfully fabricated starch–cellulose nanofibrils (CNFs) composites using twin-screw extrusion [215]. This continuous process eliminates the need for additional steps in converting raw materials to CNFs. The strong shearing force exerted during twin-screw extrusion aids in the uniform dispersion of CNFs within the starch matrix, thereby enhancing the overall uniformity and performance of the composite film. The tensile strength and modulus of the film increased with the increase in the content of CNFs, wherein the modulus and strength of the nanocomposite material increased by 1.5 times and 2.7 times, respectively, when the CNFs content was 15 wt%, compared with the unfilled starch. Hot pressing could be co-executed because it can enhance the crystallinity of the composite during the thermal pressing process, as starch molecular chains may reorganize and form a more ordered structure [216].

### 6.2. Starch-Chitosan Composites

Chitosan, a natural polymer, serves as an excellent alternative to cellulose in forming composites with starch. The incorporation of chitosan significantly enhances the tensile strength and elongation at the break of starch-based films. Research indicates that chitosan reduces the crystallinity of starch films and acts as a plasticizer, disrupting the ordered arrangement of starch molecular chains and leading to a more disordered film structure. This disruption is driven by the interaction between the amino (-NH_2_) groups in chitosan and the hydroxyl (-OH) groups in starch, forming hydrogen bonds that inhibit ordered crystallization and improve the film’s flexibility and elongation [217,218,219,220].

Other hydrophilic polysaccharides, such as carrageenan, guar gum, and xanthan gum, exhibit similar effects. These polysaccharides uniformly disperse within the starch matrix, forming interpenetrating network structures that evenly distribute stress, resulting in enhanced tensile strength and tear resistance of the composite films [221].

### 6.3. Starch–Protein Composites

Proteins, such as soy protein, corn gluten, collagen, and gelatin, are often blended with starch to produce fully biodegradable composites suitable for eco-friendly and cost-effective food packaging materials. For instance, Romani et al. demonstrated that starch–fish protein composites achieve optimal mechanical properties and water vapor permeability at a 50:50 ratio, with a tensile strength of 5.69 MPa and an elongation at break of 85.5% [222,223,224]. Similarly, edible films prepared by blending soybean protein concentrate and cassava starch with glycerin as a plasticizer exhibit improved mechanical properties when the protein content is increased to 50% and glycerin to 20%. These films outperform conventional materials like low-density polyethylene (LDPE) in tensile strength, elongation at break, and water vapor permeability [225].

The processing method also significantly impacts the properties of starch–protein composites. Solvent casting produces films with superior uniformity, transparency, and low water vapor transmission rates, whereas press molding results in composites with lower tensile strength and surface cracks. Blow molding after press molding improves density and crystallinity but may reduce expansion and tensile properties due to excessive breakdown during the process. These findings underscore the critical role of processing techniques in determining the final properties of starch–protein films [226].

### 6.4. Starch-PVA Composites

Blending starch with polyvinyl alcohol (PVA) is a common approach to fabricating composite films with enhanced mechanical properties. Starch and PVA share polar characteristics and hydroxyl groups, promoting intramolecular and intermolecular hydrogen bonds that improve compatibility and flexibility. Mao et al. reported that incorporating 9.1 wt% PVA into a starch–glycerol mixture increased tensile strength from 1.8 MPa to 4 MPa and elongation at break from 113% to 150% at 50% relative humidity. This enhancement is attributed to PVA’s ability to mitigate surface cracks and facilitate better interfacial interactions [227].

Differential scanning calorimetry (DSC) studies by Sin et al. revealed that the addition of PVA to cassava starch resulted in composites with higher initial and final transition temperatures compared to pure PVA membranes, indicating strong physical interactions. The experimental melting enthalpy of starch–PVA composites was significantly higher than the theoretical value, further evidencing robust hydrogen bond networks between the two polymers [228]. However, compatibility issues arise at higher starch contents. For example, Chen et al. observed that increasing starch content to 40 wt% led to reduced tensile strength, elongation at break, and transparency, highlighting the limitations of compatibility between starch and PVA at higher concentrations [229]. Despite these mechanical improvements, starch–PVA composites suffer from poor water barrier properties due to the abundance of hydroxyl groups in both polymers, making them highly hydrophilic. This leads to a significant reduction in tensile strength under high humidity conditions. To address this issue, various techniques have been employed, including chemical modifications of starch or PVA (e.g., grafting, cross-linking, and acid treatment) and the incorporation of nanoparticles to improve water resistance and mechanical performance [230].

Starch–hydrophilic polymer composites show great potential for sustainable applications. However, challenges such as improving water barrier properties, enhancing compatibility at high starch content, and optimizing processing methods remain. Future research should focus on developing synergistic blends, advanced chemical modifications, and novel reinforcement strategies, such as the use of nanomaterials, to further expand the applicability of starch-based composites. Table 1 provides an overview of the mechanical properties, processing methods, and performance improvements of various starch-composite films combined with different materials.

### 6.5. Coating

The application of starch film coatings is a widely used strategy to modify surface properties while simultaneously enhancing the mechanical performance of starch-based materials. Coatings improve hydrophobicity, water resistance, and interfacial adhesion, addressing some of the inherent limitations of pure starch films.

Chen et al. conducted extensive studies on using soybean oil as a coating for starch films, focusing on improving interfacial adhesion between the hydrophilic starch matrix and the hydrophobic soybean oil. In one notable study, (3-Aminopropyl) triethoxysilane (APTES) was employed as an interfacial binder due to its amphoteric functional groups. The amino group (-NH_2_) on one end of APTES can form hydrogen bonds or covalent bonds with the hydroxyl groups (-OH) on the starch film, enhancing the interaction with the starch matrix. Simultaneously, the ethoxy silane group (-Si(OC_2_H_5_)_3_) on the other end reacts with epoxy groups in acrylated epoxidized soybean oil (AESO) coatings to form siloxane bonds (Si-O-Si). This dual functionality ensures strong adhesion between the starch film and the soybean oil coating, significantly improving film durability and surface properties [231,232].

In another approach, Duan developed a highly hydrophobic and mechanically reinforced starch film by incorporating alkyl ketene dimer (AKD) into the film matrix. The inclusion of AKD significantly increased the water contact angle of the membrane, reducing its water vapor permeability and enhancing its resistance to moisture. This improvement was attributed to AKD’s ability to form a hydrophobic surface layer that repels water. Additionally, the incorporation of AKD strengthened the mechanical properties of the membrane through mechanisms such as chemical cross-linking and the formation of intermolecular hydrogen bonds. These interactions resulted in a more robust film with enhanced tensile strength and flexibility [233].

Overall, surface coatings and additives such as APTES and AKD provide effective solutions for addressing the water sensitivity and limited mechanical performance of starch films. Future research in this area could focus on developing bio-based coatings and exploring advanced cross-linking technologies to further expand the applicability of starch films in packaging and other industrial applications.

## 7. Summary

Starch films are highly valued for their biodegradability, edibility, and non-toxicity, making them attractive for a range of applications. However, their inherent brittleness remains a significant limitation to broader usage. This paper analyzed key factors from both fundamental and applied perspectives, including the resources and microstructure of starch, phase transitions during thermal processing, the use of plasticizers, chemical modification, and physical reinforcement strategies. It provided comprehensive insight into strategies and methodologies for improving the toughness of starch-based films through enumeration and comparison, objectively evaluating the advantages and shortcomings of each progress. The key findings and insights include the following:

1. Microstructures of starches have been extensively investigated at multi-scales. Generally, there are two major chemical structures: linear amylose and branch amylopectin. Amylose chains, characterized by higher flexibility and lower crystallinity, contribute to improved toughness after gelatinization and modifications. However, even high-amylose starches still fail to meet the toughness requirements for packaging films, indicating that amylose content alone is insufficient.
2. Gelatinization, which disrupts the crystalline structure of starch granules, is a prerequisite for thermal processing using traditional plastic processing techniques like extrusion and film blowing. However, gelatinized starch tends to recrystallize or retrograde, resulting in brittle materials. Addressing this brittleness remains a critical challenge.
3. Plasticizing is among the most effective strategies for improving the toughness of starch-based materials. By introducing plasticizers, the internal hydrogen bonds between starch chains are disrupted, allowing greater molecular mobility and enhancing flexibility. Ideal plasticizers should be efficient, compatible, and low in volatility, and they should perform consistently under varying environmental conditions. Water, the most common plasticizer, interacts strongly with starch but is highly volatile, resulting in unstable properties, particularly in low-humidity environments. Alternatives such as polyols (e.g., glycerol and sorbitol), saccharides (e.g., glucose and sucrose), and polar compounds (e.g., urea and citric acid) have been developed. While these alternatives improve toughness, their efficacy often diminishes under dry conditions. Emerging plasticizers like ionic liquids (ILs) and deep eutectic solvents (DESs) show promise due to their non-volatility and enhanced performance, though challenges like cost and environmental impacts persist.
4. Chemical modifications, such as esterification and etherification, can enhance starch toughness and overall performance. However, achieving high degrees of substitution (DS) while minimizing chemical residues is challenging, particularly under aqueous conditions. Extrusion has shown promise for efficient modification but introduces complexities in residue removal. Despite these advancements, modified starch films remain brittle under very low-humidity conditions.
5. Blending starch with other polymers or reinforcing it with nanomaterials like nanocellulose significantly enhances mechanical properties, including toughness. However, to preserve the benefits of full biodegradability and edibility, additives must meet strict environmental and safety criteria. While nanocellulose-reinforced starches show considerable promise, challenges such as instability under low-humidity conditions and higher production costs persist.

In conclusion, significant progress has been made in developing strategies and methodologies to improve the toughness of starch-based materials. However, a comprehensive understanding of the underlying mechanisms is still lacking, especially under low-humidity conditions. Future research should focus on unraveling these mechanisms and identifying innovative approaches to overcoming the remaining challenges.

## Figures and Tables

**Table 1:** The mechanical properties and process method of different starch-polymer composite films.

Starch	Material Composited with Starch	Additional Amount	Process Method	Performance Improvement (Compared with Pure Starch)	Reference
Cationic starch	Nanocellulose	2.0 wt %	Electrospinning	Tensile strength: +198.6%	[194]
Corn starch	Methyl cellulose	5.0 wt %	Casting	Tensile strength: +49.1%, Elongation: +124.4%	[195]
Cassava starch	Carboxymethyl cellulose	50.0 wt %	Casting	Tensile strength: +20.6%, Young’s modulus: −1.0%, Elongation: +88.9%	[197]
Cassava starch	Cellulose nanocrystals	2.0 wt %	Casting	Tensile strength: + 73.0%	[204]
Waxy maize starch	Cellulosic nanofibers	10.0 wt %	Casting	Tensile strength: +313.0%, Young’s modulus: +343.0%	[206]
Cassava starch	Lentil protein	1.5 wt %	Extrusion	Young’s modulus: +150.0%, Tensile strength: +25.0%, Elongation: −45.0%	[210]
Starch	Microcrystalline cellulose	6.0 wt%	Extrusion	Tensile strength: + 63.4%, Elongation: −71.2%	[211]
Corn starch	Oxidized cellulose fibers	15.0 wt%	Extrusion	Young’s modulus: +204.0%, Tensile strength: +352.0%	[215]
Corn starch	Chitosan	3.6 wt %	Casting	Tensile strength: +31.24 %	[217]
Corn starch	Chitosan	160.0 wt %	Casting	Elongation: + 53.9%	[218]
Cassava starch	Soy protein	50.0 wt %	Casting	Tensile strength: +24.5 %, Elongation: +111.7%	[225]
Corn starch	Polyvinyl alcohol	9.1 wt %	Extrusion	Young’s modulus: +122.2%, Elongation: +32.8%	[227]
Corn starch	Polyvinyl alcohol	10. 0 wt %	Solvent	Elongation: +230%	[230]

## Data Availability

No new data were created or analyzed in this study. Data sharing is not applicable to this article.
